# Obesity in pregnant women: a 20-year analysis of the German experience

**DOI:** 10.1038/s41430-021-00981-8

**Published:** 2021-10-26

**Authors:** Alexander Strauss, Niels Rochow, Mirjam Kunze, Volker Hesse, Joachim W. Dudenhausen, Manfred Voigt

**Affiliations:** 1 Faculty of Medicine, Christian-Albrechts-University, Kiel, Germany; 2 Department of Pediatrics, Paracelsus Medical University, General Hospital, Nuremberg, Germany; 3 Department of Pediatrics, Rostock University Medical Center, Rostock, Germany; 4 Department of Pediatrics, McMaster University, Hamilton, ON Canada; 5 Department of Gynecology and Obstetrics, University of Freiburg School of Medicine, Freiburg, Germany; 6 German Center for Growth, Development and Health Encouragement during Childhood and Youth, Berlin, Germany; 7 Department of Obstetrics, Charité University School of Medicine, Berlin, Germany; 8 Biological Anthropology, Medical Faculty, University of Freiburg, Freiburg, Germany

**Keywords:** Weight management, Epidemiology, Fat metabolism

## Abstract

**Background/objective:**

To investigate the longitudinal development of maternal body weight and analyze the influence of obesity on obstetrics during more than two decades in Germany.

**Subjects/methods:**

Data collected from the Federal state of Schleswig-Holstein (German Perinatal Survey) were analyzed with regard to the dynamics of maternal anthropometric variables (body weight, BMI) between 1995–7 and 2004–17. In total 335,511 mothers substantiated the presented study-collective. The statistical analysis was performed using IBM SPSS Statistics for Windows, Version 26.0. Armonk, NY.

**Results:**

Maternal BMI advanced significantly over the study period. Among a rise in mean periconceptional body weight (67.6–72.0 kg), the segment of obese women increased disproportionately (in average 9.4–19.2%). Despite the observed trend to late giving birth (mean maternal age 1995: 29.3 vs. 30.7 years in 2017), it was not advanced maternal age but parity that influenced the continuous increase in maternal weight (mean maternal body weight 1995–7: primi- bi-, multiparae 67.4, 68.3 and 69.0 kg vs. 2004–17: primi- bi-, multiparae 70.0, 71.5 and 73.2 kg respectively).

**Conclusion:**

Obesity is a major problem on health issues in obstetrics. Advancing maternal BMI, increasing mother’s age and derived prenatal risks considerably complicate pregnancy and delivery. It has to be emphasized that its consequences do not end with delivery or childbed, but represent a livelong burden to the mother and their offspring. Hence, multimodal strategies to reduce/control periconceptional body weight are mandatory.

## Background/objectives

In countries of high per capita income, societies are subject to rapid socioeconomic change. Wealth and nearly unlimited nutrition resources have led to the phenomenon of accelerated body growth of population in these regions. The average body length extended from generation to generation. From 1956 to 1975, the women’s average body length has increased by 10 cm. However, this effect has been stagnating ever since. Apart from adults, accelerated growth has frequently been observed with newborns with a body length of more than 55 cm: 3.4% (1986) vs. 10.1% (2001). Thereby, the anthropometric acceleration process does not only pertain measures of length but also corpulence (e.g., BMI). The prevalence of overweight and obesity has continuously increased over the past decades and has reached the significance of a pandemic health problem. This development does not exclude pregnant women and mothers. Prevalence of overweight and obesity in German mothers is 45% and 15%, respectively (2016) [1].

Maternal obesity is one of the most common risk factors for pregnancy complications (multivariate analysis): OR 1.77 (95% CI 1.28–2.45); *p* value = 0.001 [2] Periconceptional overweight/obesity are linked to a rise in complications during pregnancy such as gestational diabetes mellitus (GDM)/diabetes mellitus (OR 11.0), hypertension/preeclampsia (OR 3.6/OR 4.4), preterm birth <28 + 0 weeks (OR 2.9) [3, 4], but also an increase of perinatal risks (induced labor, postterm birth, vaginal-operative delivery, cesarean section [obesity III°: 47.4% vs. normal weight: 26.5%], anesthetic complications, wound infection and venous thromboembolism) [2, 5–9]. As far as the fetus is concerned, the following complications can be observed: fetal macrosomia (≥4.000 g) OR 1.97, 95% CI 1.88–2.06 [10, 11]; high birth weight (LGA—large for gestational age) OR 2.08, 95% CI 1.97–2.17) [12]; perinatal complications (cephalopelvic disproportion, hypoxia/asphyxia, shoulder dystocia, impacted fetus at cesarean section, neonatal injury and neonatal/postneonatal death) [7, 13–17]. For the offspring of obese mothers Whitaker additionally enumerated the relative risk of childhood obesity to be increased by 2.0 (95% CI 1.7–2.3) at 2 years of age, 2.3 (95% CI 2.0–2.6) at 3 years of age and 2.3 (95% CI 2.0–2.6) at 4 years of age [18, 19]. Long-term risks (e.g., shorter life expectancy) consecutively follow [20].

With regard to pregnancy and delivery, this context shows the predictive meaning of the mother’s periconceptional BMI for her own but also transgenerational health risks (fetal programming). From the point of view of preventive medicine, a preconceptional approximation of a normal body weight is desirable.

A body weight loss of 5–10% before pregnancy has a strongly positive effect on obese women. Moreover, it increases the chances to become pregnant in the future [21, 22].

Obesity is rampant pandemic and no subpopulations are excluded from the widespread and continuous increase of body weight in society. The aim of this study is to perform trend analyses of maternal BMI, and to evaluate its association with pregnancy and birth outcomes.

## Materials/subjects and methods

The World Health Organization (WHO) [23] and the Institute of Medicine (IOM) [24] introduced a standardized categorization of human body weight. As a convenient, broadly used comparative parameter, Body Mass Index (BMI) is established to respect not only physical weight but although the modifying influence of body composition. Body mass divided by the square of the body height (kg/m^2^) is commonly accepted to allow an unerring differentiation of underweight, normal weight, overweight and obesity. Within the overweight category according to a BMI-classification (WHO) [23], a further division into four weight-groups is to be distinguished: pre-obesity (BMI 25.0–29.9 kg/m^2^), obesity I° (BMI 30.0–34.9 kg/m^2^) obesity II° (BMI 35.0–39.9 kg/m^2^) and obesity III° (BMI ≥ 40.0 kg/m^2^) [25, 26].

In our study, we longitudinally investigated maternal weights development between 1995–7 and 2004–17 in Schleswig-Holstein (Federal State of Germany, 2,889,821 inhabitants, population density 183 inhabitants/km^2^) [27]. Study results were obtained by using the routine data collection as a part of the mandatory national Perinatal Survey of Quality Indicators. The version used (1995–2017) comprises an extensive data set containing over 160 individual items on obstetric quality assurance. This evaluation includes anthropometric measures of all birthing mothers. During 1995–7 and 2004–17, as a representative sample for Germany (and thereby exemplary for a high-income state in Europe), 335,511 pregnancies/deliveries from Schleswig-Holstein were merged and analyzed. Transferability of data is plausible by mixed federal state population characteristics: rural (38%) vs. urban (62%).

Pregnancies are followed from the first trimester until birth. The primary endpoint is the dynamic increase of maternal obesity prevalence. Secondly, the influence of parity as well as maternal age are examined. The occurrence of unfavorable maternal and fetal outcomes are put into a superordinate context.

Participant characteristics and outcomes were reported using descriptive statistics. The data were tested for normal distribution using the Kolmogorov–Smirnov test. Parametric and nonparametric tests were employed accordingly. The trend of change in body weight, BMI, and maternal age during the years 1995–7 and 2004–17 was analyzed using regression analysis. The year-chronological evaluation regarding maternal body weight development data was compared with the Mann–Whitney U test regarding parity, BMI-allocation, and mothers age. BMI was categorized into normal weight and obese. The parity was categorized into nullipara, one and two or more previous deliveries. For categorical variables, odd ratios were analyzed. The level of significance was defined with a *p* < 0.05. The statistical analysis was performed using IBM SPSS Statistics for Windows, Version 26.0. Armonk, NY.

## Results

**Fig. 1:**
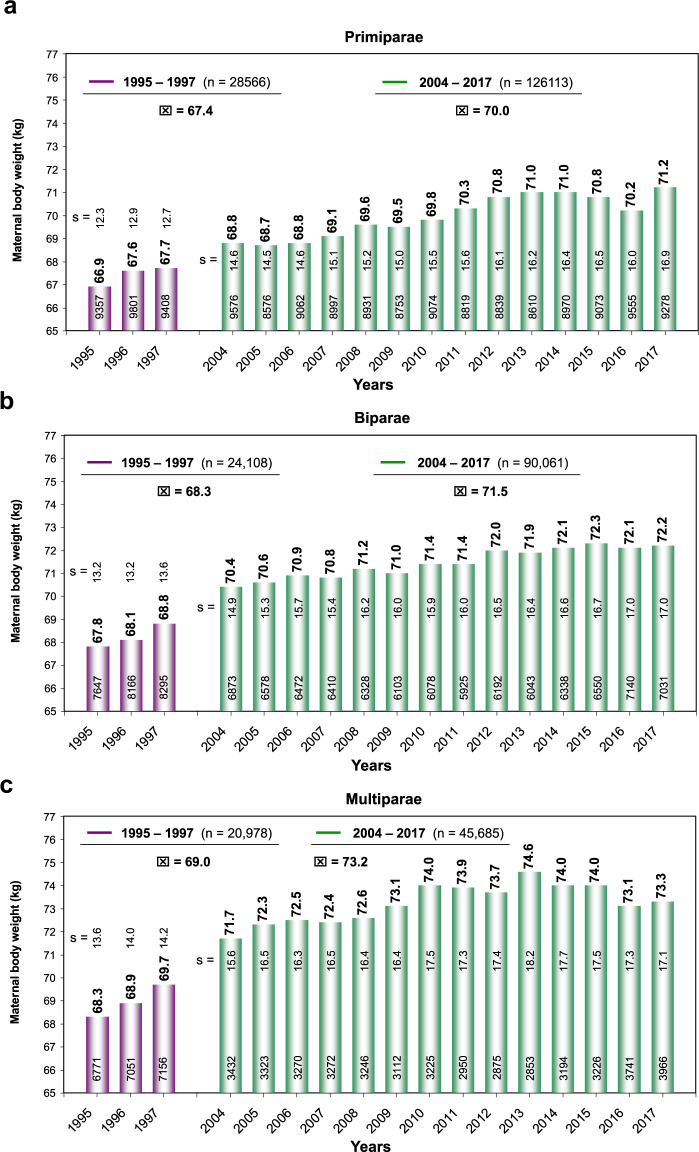
Maternal body weight. Longitudinal observation of maternal body weight (mean and standard deviation [s]) at the first obstetrical visit (proof of pregnancy) in Schleswig-Holstein of the periods 1995–7 and 2004–17 (**a:** Primiparae, **b:** Biparae, **c:** Multiparae).

**Fig. 2:**
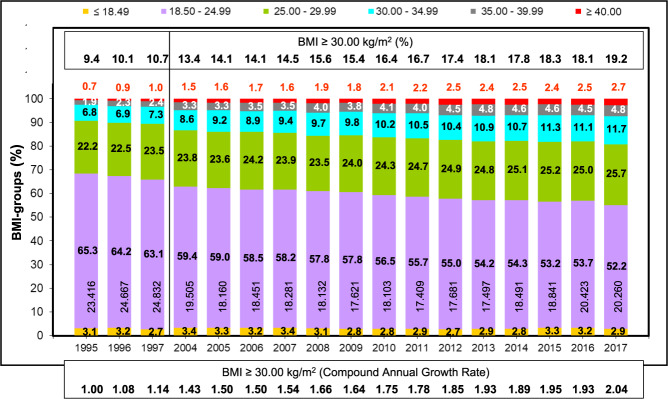
Preconceptional BMI. Development of the BMI repartition of pregnant women in Schleswig-Holstein (1995–7 and 2004–17) according to the WHO body weight classification of adults (underweight, normal weight; pre-obesity, obesity I°, II° und III°): significant increasing percentage and Odds ratio for maternal obesity.

**Fig. 3:**
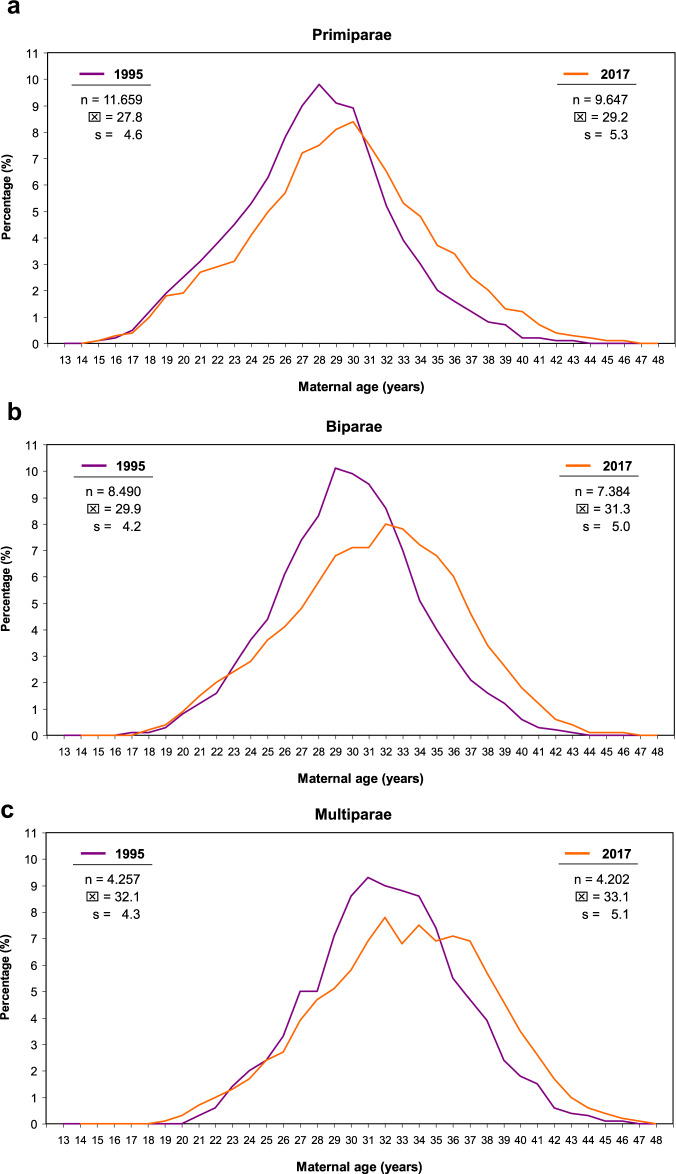
Maternal age and paritiy. Change of maternal age distribution in Schleswig-Holstein between 1995 (purple) and 2017 (orange) depending on parity (**a:** Primiparae, **b:** Biparae, **c:** Multiparae).

**Fig. 4:**
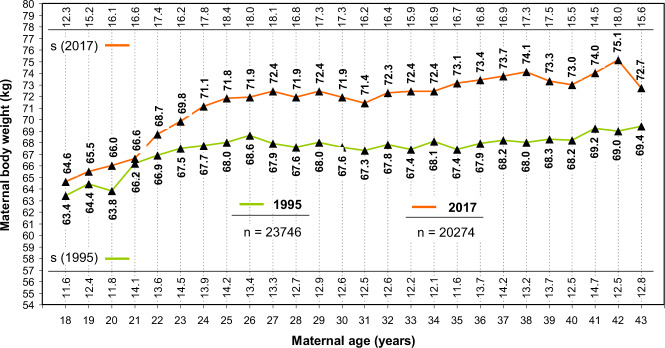
Body weight and mothers age. Differences in periconceptional maternal body weight (mean and standard deviation [s]) according to maternal age in Schleswig-Holstein). The purple curve shows weight distribution in correlation to maternal age (years) in 1995 (*n* *=* 23,746), the orange curve gives the corresponding information for 2017 (*n* *=* 20,274).

Over the past decades, there has been a significant overall increase in the average body weight and maternal age in high-income countries. Periconceptional maternal anthropometric dimensions (e.g., body height and body weight) are recorded (Perinatal Basic-Questionnaire) at women’s first obstetrical visit during the first trimester (proof of pregnancy). Between 1995–7 and 2004–17 an almost linear increase of the maternal weight (overall study group [*n* = 335.511]: 1995 – 67.6 kg and to 2017 – 72.0 kg) can be noticed (*p* < 0.001). With respect to increasing parity, this progress becomes even more obvious (1995 and 2017: weight increase of 4.3, 4.4 and 5.0 kg for primi-, bi- and multiparous women respectively). This even more, as also the starting body weights raise with parity in a disproportionate way (mean 69.5 kg (*n* = 154,679), 70.8 kg (*n* = 114,169) and 71.9 kg (*n* = 66,663) for primi-, bi- and multiparous women respectively) (Fig. 1). Next to that, there is a significant increase in prevalence of obesity I°–III° from 9.4% (2201/23416) to 19.2% (3890/20260) (*p* < 0.001) going along with an inversely decreasing prevalence of normal periconceptional BMI from 65.3% (15,291/23,416) in 1995 to 52.2% (10,576/20,260) in 2017 respectively (Fig. 2). Between 1995–7 and 2004–17 not only the maternal weight, but also pregnant women’s age have been subject to a continuous increase. Therefore, logical evidence can be attached to the respective increases. In 1995, the average mother’s age when giving birth to her first, second and ≥third child in Schleswig-Holstein (Germany) was 27.8, 29.9, and 32.1 years respectively. Up to 2017 these rates increased by 1.4, 1.4 and 1.0 years (Fig. 3) (*p* < 0.001). In particular the birth rate of 40–44-year-old mothers nearly tripled (OR 2.9) during the study period (1.6% in 1995 to 4.7% in 2017). Following the hypothesis of interdependency concerning the observed rise of both, body weight and maternal age, these parameters were set in relation leading to the following insights (Fig. 4):

- Maternal obesity significantly rose during investigation period: mean 4.4 kg for all study participants (*p* < 0.001)
- Maternal age substantially increased over the study phase (mean 1.4 years [median 2.0 years])
- Maternal weight differences between 1995 and 2017 were independent from maternal age (BMI-increase was consistent in all maternal age groups, apart from mothers on the lower end of the reproductive age spectrum: <23 years (*p* < 0.001)

## Discussion

Obesity as a universal health factor has considerable prognostic relevance for our society. Once considered a problem only in high-income countries, today increasing body weight is also on the rise in low- and middle-income countries, particularly in urban settings.

The steady rise of maternal body weight in the US exceeds by far the European progression in quantity and dynamic [28]. Obesity (BMI *>* 29.9 kg/m^2^) in pregnancy: US 14% → 30% (1997–2007) vs. Germany 8% → 14% (1990–2009). Apart from different levels of absolute numbers, the presented study reflects the big picture observable in Northern America. Doubling times and thus dynamics of the increase of maternal obesity on both sides of the Atlantic Ocean correspond to each other and thereby predict the upcoming challenges not only for obstetrics.

In the past decades Central European industrial countries’ population has gone through an increase of body mass among its citizens. In Germany, women of normal weight at reproductive age (18–50 years) put on 0.28 kg/year on average. In comparison, the average increase of body weight of 1.13 kg/year with obese women of the same age is much higher. Summarily the rise of body weight in the focused period of life results in 9 kg and up to 36 kg in cases of obesity [29]. Elevated BMI-parameters and the central type of fat tissue repartition strain periconceptional women evermore. (Table 1). In 2017, the periconceptional body weight of 44.9% of pregnant women in Schleswig-Holstein exceeded the BMI of 24.9 kg/m^2^. 19.2% were obese (BMI ≥ 30.0 kg/m^2^) and 2.7% faced third-grade obesity (BMI ≥ 40.0 kg/m^2^). The negative relationship between advanced maternal body weight and unfavorable outcomes of pregnancy is common knowledge (e.g., maternal obesity → fetal programming). Maternal obesity can result in both pregnancy and delivery complications for mother and child (e.g., placental insufficiency, stillbirth, fetal malformations). Furthermore and according to Barker’s hypothesis, overweight and obesity are major risk factors for a number of chronic diseases, including diabetes, cardiovascular diseases and cancer [30]. Moreover obesity accounts for a dose-dependent rise in maternal mortality (OR 1.29–1.39/5 kg/m^2^) [31]. Despite compliance to established weight control protocols, morbidity and mortality remain elevated on the long term. Besides the short-and long-term risks for the mother, obesity in pregnancy affects the fetus. In infancy but also in later life, transgenerational detrimental sequelae are to be expected.

**Table 1:** Prevalence of pre-obesity, obesity I–II° and obesity III° in German women of childbearing age [45, 46].

	BMI 25.0–29.9 kg/m^2^ % (95% CI)	BMI 30.0–39.9 kg/m^2^ % (95% CI)	BMI ≥ 40.0 kg/m^2^ % (95% CI)
18–29 years	30.0 (25.9–34.5)	9.6 (7.2–12.7)	0.9 (0.3–2.7)
30–39 years	38.0 (32.8–43.5)	17.9 (14.0–22.7)	2.3 (1.1–4.6)
40–49 years	46.4 (42.1–50.8)	18.6 (15.6–22.2)	2.1 (1.2–3.6)

Obese mothers‘ offspring faces an increase of health burden (e.g., cardiovascular diseases, diabetes mellitus, dyslipidemia) and a generally shortened life expectancy. Especially metabolic syndrome at any time up to 11 years is to be expected in 50% of children in obese mothers additionally suffering from GDM and 29% in non-diabetic pregnant women [14, 32].

Numerous publications support the evidence that late maternity is associated with various risks to the mother and hazards to perinatal outcomes [33–38]. Over the past two decades, there has been not only a rising proportion of deliveries among mothers with advanced age but also with increased body weight. Looking at a long period of time (1995–7 and 2004–17), the presented data demonstrate a continuous increase in average maternal weight and BMI according to pre-obesity and obesity I°–III°. This observation is at the expense of a decrease of normal periconceptional BMI. Our results analyze interdependent effects of mother’s age and parity when the progressing weight distribution of pregnant women is concerned. The lowest BMI-increase-rates were found in primiparous women. Therefore, multiparity augments most strongly the incidence of obesity over time. The presented data characterize this observation to be unbiased by the raise of maternal weight irrespective to the fact, that mothers studied after their first or a further delivery do not reach again their original body weight (Fig. 1).

Dependency of increasing maternal weight in pregnant women and increasing age is already discussed for quite a long time. Against commonly communicated opinion [25, 39–42], the presented data from Schleswig-Holstein contradict that advanced maternal age does reach significant impact on the coincidently observed increase of maternal BMI. Rather obesity in pregnancy escalates independently from mothers’ age.

Limitations to study results following from regional differences between federal states in Germany can be balanced when cross-regional significance is discussed. Nevertheless, by interpretation of social, political and medical fundamentals, as well as a comparison of obstetrical outcome data from the German Perinatal Survey substantial variations to other German regions can be ruled out [43, 44]. This is mainly due to the survey sample size and the homogeneous distribution ratios of study participants. In addition, the deviation of the regular relation between age and body weight evolution at both ends of the study collective can be explained by a reduced survey sample size in these age groups. As it is a common limit to population-based surveys, the retrospective character of data acquisition, inevitable in a national survey, has to be considered. However, unavoidable single mistakes with the data registration and input do not establish evidence of a systematic selection bias. The use of the BMI for anthropometric classification represents a clinically established routine procedure with the known methodological constraints. In addition, the extent of collateral confounders such as maternal constitution, fat tissue distribution, or body height needs to be kept in mind.

## Conclusion

Considering the past decades, a future accelerating increase in maternal weight followed by rapidly expanding numbers of obese mothers is on the rise. Answers to medical questions on etiology, dynamics and the consequences of increasing maternal body weight offer the opportunity to establish intervention strategies for the future. Both the societal context and suggested medical solutions have to become the focus of attention. Contrary to the expectation pregnant women of all age groups are similarly affected by obesity risks. Therefore prevention can not be limited to certain “risk groups”. The need for action is urgent because of the transgenerational far-reaching consequences. Successful maternal weight control is thus of paramount importance to the mother as well as their descendants.

Ongoing increase in the average maternal age as well as rising body weight/proportion of obesity will continue to challenge obstetrics also in the coming years. Nonetheless, both effects will influence modern perinatology more by summation than by multiplication. Physical activity, diet adjustment or in distinctive cases surgery are options to interrupt the cascade of deleterious effects. Professional physical support in combination with practice-oriented health information focused on mother and child has to begin/take place preconceptionally. Obstetrics offers the appropriate time and instruments to influence the fate of all—mother, child, and grandchildren. In this respect, and because of the numerous maternal and fetal risks associated with obesity, campaigns focusing on the societal awareness especially on the long term consequences of obesity are mandatory.
